# Impaired vascular relaxation in type 2 diabetes: A systematic review and meta-analysis

**DOI:** 10.17179/excli2024-7330

**Published:** 2024-07-09

**Authors:** Sajad Jeddi, Zahra Bahadoran, Parvin Mirmiran, Khosrow Kashfi, Asghar Ghasemi

**Affiliations:** 1 Endocrine Physiology Research Center, Research Institute for Endocrine Sciences, Shahid Beheshti University of Medical Sciences, Tehran, Iran; 2 Nutrition and Endocrine Research Center, Research Institute for Endocrine Sciences,Shahid Beheshti University of Medical Sciences, Tehran, Iran; 3 Department of Molecular, Cellular, and Biomedical Sciences, Sophie Davis School of Biomedical Education, City University of New York School of Medicine, NY, USA

**Keywords:** Type 2 diabetes, vascular complications, vascular smooth muscle, acetylcholine, methacholine, sodium nitroprusside, glyceryl trinitrate

## Abstract

Type 2 diabetes (T2D) significantly increases the risk of vascular complications (12-32 %), which are a major cause of death (over 50 %) in T2D patients. In T2D, both endothelial (ET) and vascular smooth muscle (VSM) cells are impaired, which act as independent risk factors for cardiovascular disease. Thus, the question of this systematic review and meta-analysis is: Do ET-dependent and -independent VSM relaxation impair in T2D? We systematically searched PubMed and Scopus databases until March 2024; 44 eligible clinical trial studies (68, 16, 30, and 50 study arms for acetylcholine (ACh), methacholine (MTH), sodium nitroprusside (SNP), and glyceryl trinitrate (GTN)) published were included. ET-dependent VSM relaxation in response to ACh (overall ES = -28.9 %, 95 % CI: -35.2, -22.7; p<0.001) and MTH (overall ES = -55.3 %, 95 % CI: -63.6, -47.1; p<0.001) decreased in T2D patients compared to controls. ET-independent VSM relaxation in response to SNP (overall ES = -17.2 %, 95 % CI: -35.2, -22.7; p<0.001) and GTN (overall ES = -63.2 %, 95 % CI: -81.0, -45.5; p<0.001) decreased in T2D patients compared to controls. Our meta-analysis showed reductions in both ET-dependent (~40 %) and ET-independent (~25 %) VSM relaxation. The decrease was more pronounced for MTH (~55 %) compared to ACh (~30 %) and for GTN (~63 %) compared to SNP (~17 %). These findings suggest that dysfunction of both ET and VSM contributes to impaired VSM relaxation in T2D patients.

See also the graphical abstract(Fig. 1).

## Introduction

Globally, over 536 million adults (10 %) had type 2 diabetes (T2D) in 2021, and it is estimated that this number will rise to 783.2 million (12 %) by 2045 (Sun et al., 2022[72]). Patients with T2D have a 12-32 % risk of vascular complications (Einarson et al., 2018[21]; Aikaeli et al., 2022[2]), which increase cardiovascular morbidity and mortality by 2-4 times compared to those without T2D (Creager et al., 2003[18]; Lüscher et al., 2003[48]) and are responsible for over 50 % of deaths in T2D patients (Einarson et al., 2018[21]). In T2D, both endothelial and vascular smooth muscle (VSM) cells are impaired, which act as independent risk factors for cardiovascular diseases (CVD) in T2D (Ganz and Vita, 2003[24]; Lerman and Zeiher, 2005[46]; Kawano et al., 2012[40]). 

Endothelium (ET) produces several vasoactive substances to maintain vascular homeostasis (Park and Park, 2015[58]), of which nitric oxide (NO) is the most important (Kelm, 1999[42]). ET produces about 40-60 % of the whole-body NO (i.e., 1100 μmol/day) (Ghasemi and Jeddi, 2022[27]), with over 60 % of this NO transferred to neighboring VSMs in the blood vessel wall (Malinski et al., 1993[50]). Vascular NO resistance in T2D is characterized by decreased NO production by ET, increased NO inactivation, and impaired responsiveness of VSMs to vasodilatory effects of NO (Bahadoran et al., 2023[5]). Vascular NO resistance, independent of other well-known risk factors (Schächinger et al., 2000[65]; Halcox et al., 2002[31]), is associated with future cardiovascular events such as heart attack and CVD mortality (Bahadoran et al., 2023[5]). Understanding the link between VSM dysfunction and NO is important for developing strategies to reduce the risk of life-threatening events.

We recently reviewed the evidence for and the underlying mechanisms of impaired vascular relaxation in animals and humans with T2D (Bahadoran et al., 2023[5]). Acetylcholine (ACh) and methacholine (MTH)-related VSM relaxation decreased in T2D (Caballero et al., 1999[14]; Brooks et al., 2008[10]) by ~13-94 % (Bahadoran et al., 2023[5]). However, VSM relaxation in responses to sodium nitroprusside (SNP) and glyceryl trinitrate (GTN) in T2D is controversial; some studies report decreases of 6-42 % (Natali et al., 2006[54]; Beer et al., 2008[7]), and others show preserved (Steinberg et al., 1996[70], 2000[71]) or even increased relaxation (Goodfellow et al., 1996[29]; Steinberg et al., 1996[70]). A meta-analysis conducted in 2013 reported that ET-independent VSM relaxation significantly decreased in T2D patients compared with controls [standardized mean difference (SMD) = −0.68; 95 % confidence interval (CI) −0.84, −0.52)] (Montero et al., 2013[52]). However, this analysis did not consider several factors: ET-dependent vasodilation (ACh and MTH-related VSM relaxation), separate main effects and dose-response analyses of each vasodilator (ACh, MTH, SNP, and GTN), and the duration of T2D. ET-dependent (in response to ACh and MTH) and ET-independent (in response to SNP and GTN) VSM relaxation in T2D are influenced by the type of vasodilators used and the duration or severity of the disease (Bahadoran et al., 2023[5]). Thus, the question of this systematic review and meta-analysis is: Do endothelium-dependent and -independent VSM relaxation impair in patients with T2D? We also assess the association between vasodilation and duration of diabetes.

## Materials and Methods

### Search strategy and study selection 

We followed the Preferred Reporting Items for Systematic Reviews and Meta-Analyses (PRISMA) 2020 updated guideline (Page et al., 2021[57]) to design and perform this study and analyze and report the results. Databases (PubMed and Scopus) were searched until March 2024 using the combination of the following search terms in Title/Abstract: (Diabetes) AND [(acetylcholine) OR (methacholine) OR (nitroglycerin) OR (sodium nitroprusside) OR (glyceryl trinitrate) OR (trinitroglycerin) OR (endothelium-dependent vascular relaxation OR (endothelium-independent vascular relaxation OR (forearm blood flow OR (brachial artery)].

**Figure 2:**
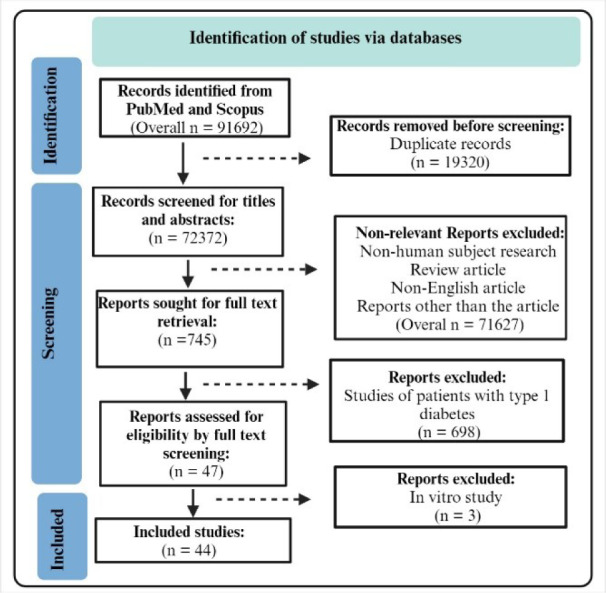
PRISMA flow diagram of the included studies. Created with BioRender.com

The records were extracted from each database to Endnote version 18 software, and the duplicate reports were removed. The first screening step evaluated titles and abstracts to exclude irrelevant articles. Excluded articles were studies on non-human subjects, studies not in English, review articles (including systematic reviews and meta-analyses), and other report types besides journal articles (e.g., conference abstracts, comments, letters to the editor, and books). Full texts of the relevant studies were then sought and screened cautiously and thoroughly for their eligibility, and studies on type 1 diabetes were excluded. All publications assessed the effect of ET-dependent and -independent VSM relaxation in T2D patients were included. Eventually, eligible studies (44 studies with 68, 16, 30, and 50 study arms for ACh, MTH, SNP, and GTN, respectively) were considered for final inclusion (Figure 2(Fig. 2)). The references of the final included studies were also screened to reduce the risk of missing relevant studies.

### Data extraction 

Information extracted included the first author's name, year of publication, age, sex (% of men), sample size, duration of T2D, vascular region assessed, and dose of vasodilators used. The mean and standard deviation (SD) of response to vasodilators were also extracted. If a study had measured response to vasodilators following several doses and/or durations of T2D, each measurement was considered a separate experimental study. These data were extracted from the article texts or tables; if a study had represented the results only as a graph, the data was extracted by the Adobe Photoshop software using a previously described method (Gheibi et al., 2019[28]). 

### Statistical analysis

The Weighted Mean Difference (WMD) was used as the primary outcome for measuring the difference in VSM relaxation in response to ET-dependent (ACh and MTH) and ET-independent (SNP and GTN) vasodilators. WMD was used as the effect size (ES) instead of SMD because all included studies reported the vasodilatory effect of ACh, MTH, SNP, and GTN as a percentage change. For studies lacking the percentage change data, it was calculated from the reported raw data.

The meta-analysis was performed after separating ACh, MTH, SNP, and GTN data. STATA version 17 was used for statistical analysis and making illustrations. The random-effect model was chosen instead of the fixed-effect model due to its suitability when significant heterogeneity (variation) exists between studies. The WMD was analyzed and reported separately with a 95 % CI for each vasodilator (ACh, MTH, SNP, and GTN). Two-sided *P*-values<0.05 and between 0.05 and 0.10 were considered statistically and marginally significant, respectively.

Heterogeneity was assessed using I² (I-squared), which quantifies the proportion of variation in effect sizes attributable to factors beyond sampling error (percentage of heterogeneity indicates the extent of the studies that the CI of their outcome does not overlap with the overall ES), and Cochran's Q test (test of homogeneity), which statistically evaluates the likelihood that the observed differences in study outcomes are due to chance and representing the probability that the outcomes of the studies are homogenous (Higgins et al., 2003[35]). The results of I² are reported and interpreted according to the established categories: 0-25 % = no significant heterogeneity, 25-50 % = low heterogeneity, 50-75 % = moderate heterogeneity, and >75 % = high heterogeneity (Higgins et al., 2003[35]). The P-value is reported for Cochran's Q test, and a value less than 0.1 typically suggests significant heterogeneity. Publication bias was evaluated using Egger's regression test and funnel plots (Egger et al., 1997[20]). A funnel plot is a scatter plot of the intervention effect estimates from individual studies (X-axis) against the standard error of the estimated effect (Y-axis). Egger's test examines potential publication bias in a meta-analysis by examining funnel plot asymmetry. Specifically, Egger's test involves a linear regression of the intervention effect estimates on their standard errors, weighted by their inverse variance (Egger et al., 1997[20]).

Random effects meta-regression was performed to explore potential sources of heterogeneity among the included studies. This analysis examined the dose of each vasodilator (ACh, MTH, SNP, and GTN) and the duration of T2D as possible influencing factors. For meta-regression, the R^2^ was reported, indicating the amount of heterogeneity explained by the moderator; if the dose of vasodilators and duration of T2D were not available for a study, the study was excluded from the meta-regression analysis. Additionally, subgroup analysis was conducted based on the type of vessels studied to determine if the impairment varied depending on the specific vessel type (Yugar-Toledo, 2004[85]). 

Variability in vasodilator responses across studies was observed due to differences in the administered doses and the duration of the study. To address this variation and provide a more precise evaluation of the studied vasodilators' effects, separate dose-response analyses were conducted for each vasodilator, including ACh, MTH, SNP, and GTN. 

## Results

### Characteristic of studies: ET-dependent VSM relaxation 

Twenty-two studies assessed the effect of ACh on VSM relaxation in T2D patients (McVeigh et al., 1992[51]; Watts et al., 1996[80]; Avogaro et al., 1997[3]; Hogikyan et al., 1998[36]; Caballero et al., 1999[14]; Gazis et al., 1999[26]; Vavuranakis et al., 1999[77]; Butler et al., 2000[13]; Heitzer et al., 2000[33]; Preik et al., 2000[62]; Kingwell et al., 2003[44]; Pistrosch et al., 2004[60]; Vehkavaara and Yki-Järvinen, 2004[78]; Natali et al., 2006[54]; Woodman et al., 2006[83]; Schmiedel et al., 2007[66]; Sivitz et al., 2007[68]; Beer et al., 2008[7]; Brooks et al., 2008[10]; Yeh et al., 2009[84]; Chousos et al., 2010[16]; Bock et al., 2021[8]). These studies involved 664 patients with T2D and 415 control subjects. The mean age of T2D patients in these studies was 56.2±4.9 years with a range of 42 (Avogaro et al., 1997[3]) to 65 (Butler et al., 2000[13]) years, and the mean age of control subjects was 53.8±5.8 years with a range of 38 (Avogaro et al., 1997[3]) to 58 (Schmiedel et al., 2007[66]) years. Nineteen studies included both sexes (McVeigh et al., 1992[51]; Hogikyan et al., 1998[36]; Caballero et al., 1999[14]; Gazis et al., 1999[26]; Vavuranakis et al., 1999[77]; Butler et al., 2000[13]; Heitzer et al., 2000[33]; Preik et al., 2000[62]; Pistrosch et al., 2004[60]; Vehkavaara and Yki-Järvinen, 2004[78]; Natali et al., 2006[54]; Woodman et al., 2006[83]; Schmiedel et al., 2007[66]; Sivitz et al., 2007[68]; Beer et al., 2008;[7] Brooks et al., 2008[10]; Yeh et al., 2009[84]; Chousos et al., 2010[16]; Bock et al., 2021[8]), and three studies were conducted in men (Watts et al., 1996[80]; Avogaro et al., 1997[3]; Kingwell et al., 2003[44]). The mean duration of T2D was 6.5±2.4 years, with a range of 3.6 (Watts et al., 1996[80]) to 10.8 (Beer et al., 2008[7]) years. The ACh was administered in the brachial artery (McVeigh et al., 1992[51]; Watts et al., 1996[80]; Avogaro et al., 1997[3]; Hogikyan et al., 1998[36]; Gazis et al., 1999[26]; Vavuranakis et al., 1999[77]; Butler et al., 2000[13]; Heitzer et al., 2000[33]; Preik et al., 2000[62]; Pistrosch et al., 2004[60]; Vehkavaara and Yki-Järvinen, 2004[78]; Natali et al., 2006[54]; Woodman et al., 2006[83]; Schmiedel et al., 2007[66]; Sivitz et al., 2007[68]; Yeh et al., 2009[84]; Bock et al., 2021[8]), femoral artery (Kingwell et al., 2003[44]), and forearm skin microcirculation (FMS) (Caballero et al., 1999[14]; Beer et al., 2008[7]; Brooks et al., 2008[10]; Chousos et al., 2010[16]). The ACh dose range used was 0.45 (Natali et al., 2006[54]) and 35 (McVeigh et al., 1992[51]) µg/min. Characteristics of studies with more details are provided in Table 1(Tab. 1) (References in Table 1: Avogaro et al., 1997[3]; Beckman et al., 2010[6]; Beer et al., 2008[7]; Bock et al., 2021[8]; Brooks et al., 2008[10]; Butler et al., 2000[13]; Caballero et al., 1999[14]; Chousos et al., 2010[16]; Gazis et al., 1999[26]; Heitzer et al., 2000[33]; Hogikyan et al., 1998[36]; Kingwell et al., 2003[44]; McVeigh et al., 1992[51]; Natali et al., 2006[54]; Pistrosch et al., 2004[60]; Preik et al., 2000[62]; Schmiedel et al., 2007[66]; Sivitz et al., 2007[68]; Steinberg et al., 1996[70], 2000[71]; Ting et al., 1996[74]; Vavuranakis et al., 1999[77]; Vehkavaara and Yki-Järvinen, 2004[78]; Watts et al., 1996[80]; Williams et al., 1996[81]; Woodman et al., 2006[83]; Yeh et al., 2009[84]). 

The effect of MTH on VSM relaxation in T2D patients has been reported in five studies (Steinberg et al., 1996[70]; Ting et al., 1996[74]; Williams et al., 1996[81]; Steinberg et al., 2000[71]; Beckman et al., 2010[6]). These studies involved 60 patients with T2D and 105 control subjects. The mean age of T2D patients in these studies was 42.1±5.5 years with a range of 36 (Steinberg et al., 2000[71]) to 53 (Beckman et al., 2010[6]) years, and the mean age of control subjects was 38.5±4.9 years with a range of 34 (Schächinger et al., 2000[65]) to 44 (Beckman et al., 2010[6]) years. Three studies included both sexes (Ting et al., 1996[74]; Williams et al., 1996[81]; Beckman et al., 2010[6]), one study was conducted in men (Steinberg et al., 2000[71]), and one study did not specify sex (Steinberg et al., 1996[70]). The duration of T2D was reported only in 2 studies (3.7 (Ting et al., 1996[74]) and 4 (Williams et al., 1996[81]) years). The MTH was administered in brachial artery (Ting et al., 1996[74]; Williams et al., 1996[81]; Beckman et al., 2010[6]), femoral artery (Steinberg et al., 2000[71]), and FMS (Steinberg et al., 1996[70]). The MTH dose range used was 0.3 (Beckman et al., 2010[6]) and 15 (Steinberg et al., 2000[71]) µg/min.

### Meta-analyses: ET-dependent VSM relaxation 

**Figure 3:**
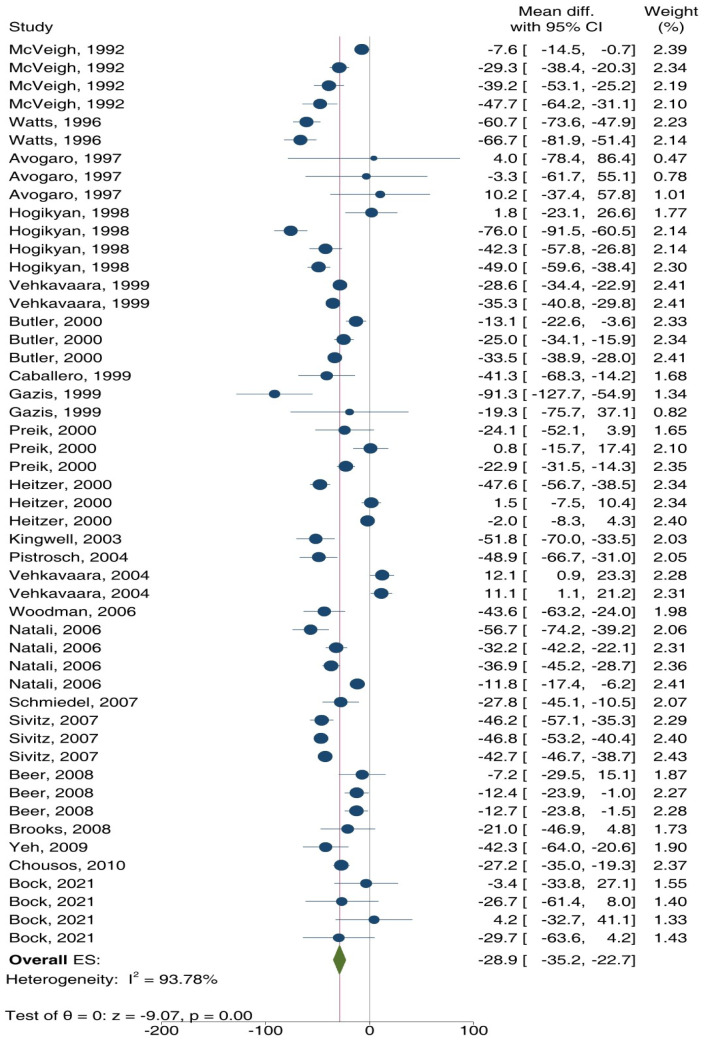
Overall effect size (ES) of endothelium (ET)-dependent vascular smooth muscle (VSM) relaxation in response to acetylcholine (ACh) in patients with type 2 diabetes (T2D) compared to controls. CI, confidence interval

**Figure 4:**
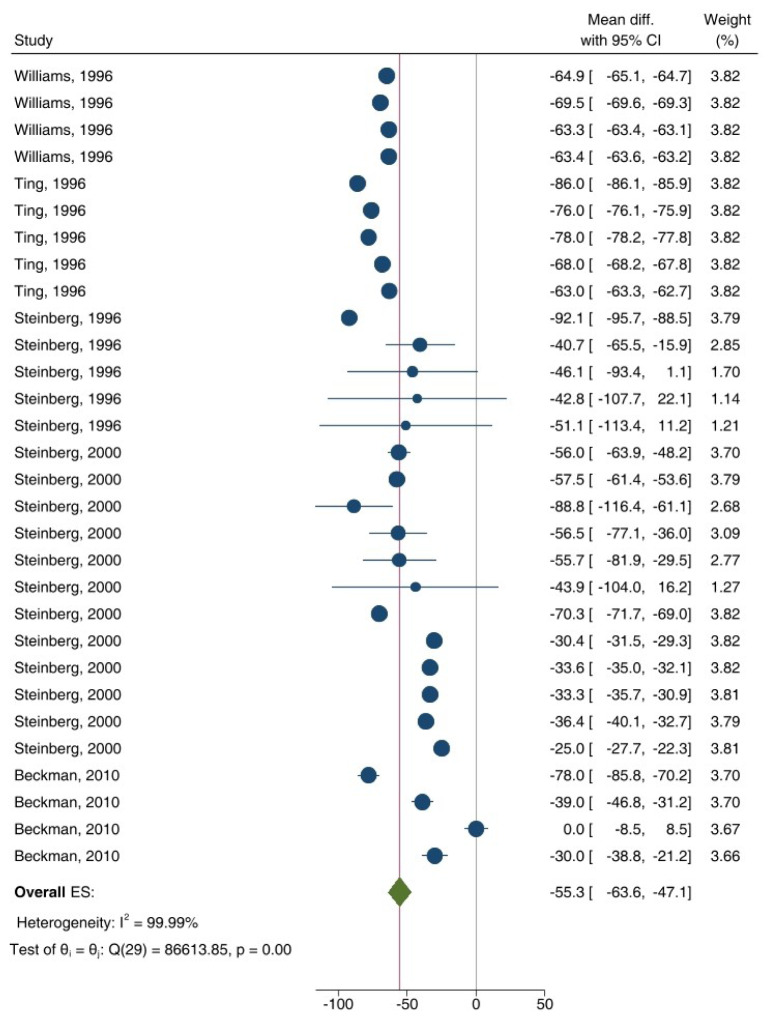
Overall effect size (ES) of endothelium (ET)-dependent vascular smooth muscle (VSM) relaxation in response to methacholine (MTH) in patients with type 2 diabetes (T2D) compared to controls. CI, confidence interval

As shown in Figures 3(Fig. 3) and 4(Fig. 4), ET-dependent VSM relaxation in response to ACh (overall ES = -28.9 %, 95 % CI: -35.2, -22.7; p<0.001) (Figure 3(Fig. 3); References in Figure 3: Avogaro et al., 1997[3]; Beer et al., 2008[7]; Bock et al., 2021[8]; Brooks et al., 2008[10]; Butler et al., 2000[13]; Caballero et al., 1999[14]; Chousos et al., 2010[16]; Gazis et al., 1999[26]; Heitzer et al., 2000[33]; Hogikyan et al., 1998[36]; Kingwell et al., 2003[44]; McVeigh et al., 1992[51]; Natali et al., 2006[54]; Pistrosch et al., 2004[60]; Preik et al., 2000[62]; Schmiedel et al., 2007[66]; Sivitz et al., 2007;[68] Vavuranakis et al., 1999[77]; Vehkavaara and Yki-Järvinen, 2004[78]; Watts et al., 1996[80]; Woodman et al., 2006[83]; Yeh et al., 2009[84]) and MTH (overall ES = -55.3 %, 95 % CI: -63.6, -47.1; p<0.001) (Figure 4(Fig. 4); References in Figure 4: Beckman et al., 2010[6]; Steinberg et al., 1996[70], 2000[71]; Ting et al., 1996[74]; Williams et al., 1996[81]) decreased in T2D patients compared to controls. In addition, high heterogeneity was observed in this analysis, indicated by I² values of 93.8 % (p<0.001) and 99.9 % (p<0.001) for ACh and MTH, respectively. Overall, ET-dependent VSM relaxation in response to ACh/MTH in T2D patients decreased by 39.2 % (95 % CI: -46.0, -33.5; p<0.001).

### Meta-regression, subgroup analysis, and publication bias: ET-dependent VSM relaxation 

ET-dependent VSM relaxation in response to ACh has not been affected by the dose of ACh (p=0.875, B = -0.064; Supplementary Figure 1A) and the duration of T2D (p=0.198, B = 1.96; Supplementary Figure 1B). In contrast, ET-dependent VSM relaxation in response to MTH affected by dose of MTH (p=0.007, B = 2.25; Supplementary Figure 1C) and duration of T2D (p= 0.058, B = 29.84; Supplementary Figure 1D); higher doses of MTH and longer duration of T2D were associated with a lower WMD (lower impairment in response to MTH) of VSM relaxation between T2D patients and controls. 

The test of group differences was statistically significant between the subgroups for the type of vessels in studies that used ACh (p=0.004) but not for MTH (p=0.266) (Table 2(Tab. 2)). 

The funnel plot for the mean difference in the VSM function of studies included in the meta-analysis was asymmetrical (Supplementary Figure 2A for ACh and 2B for MTH). However, Egger's test detected that the observed asymmetry was not due to publication bias (p=0.529, p=0.504 for ACh and MTH, respectively).

### Characteristic of studies: ET-independent VSM relaxation 

Twenty-eight studies assessed the effect of SNP on VSM relaxation in T2D patients (Steinberg et al., 1996[70]; Ting et al., 1996[74]; Watts et al., 1996[80]; Williams et al., 1996[81]; Avogaro et al., 1997[3]; O'Brien et al., 1997[55]; Hogikyan et al., 1998[36]; Caballero et al., 1999[14]; Gazis et al., 1999[26]; Vavuranakis et al., 1999[77]; Butler et al., 2000[13]; Preik et al., 2000[62]; Steinberg et al., 2000[71]; Van de Ree et al., 2001[75]; Van Etten et al., 2002[76]; Kingwell et al., 2003[44]; Vehkavaara and Yki-Järvinen, 2004[78]; Natali et al., 2006[54]; Woodman et al., 2006[83]; Schmiedel et al., 2007[66]; Sivitz et al., 2007[68]; Sokolnicki et al., 2007[69]; Beer et al., 2008[7]; Brooks et al., 2008[10]; Yeh et al., 2009[84]; Chousos et al., 2010[16]; Shemyakin et al., 2012[67]; Mahdi et al., 2018[49]). These studies involved 724 patients with T2D and 519 control subjects. The mean age of T2D patients in these studies was 54.6±7.4 years with a range of 36 (Steinberg et al., 2000[71]) to 53 (Beckman et al., 2010[6]) years, and the mean age of control subjects was 51.7±8.2 years with a range of 28 (Steinberg et al., 2000[71]) to 66 (Schmiedel et al., 2007[66]) years. Nineteen studies included both sexes (Ting et al., 1996[74]; Williams et al., 1996[81]; Hogikyan et al., 1998[36]; Caballero et al., 1999[14]; Gazis et al., 1999[26]; Vavuranakis et al., 1999[77]; Butler et al., 2000[13]; Preik et al., 2000[62]; Van Etten et al., 2002[76]; Vehkavaara and Yki-Järvinen, 2004[78]; Natali et al., 2006[54]; Woodman et al., 2006[83]; Schmiedel et al., 2007[66]; Sokolnicki et al., 2007[69]; Beer et al., 2008[7]; Brooks et al., 2008[10]; Yeh et al., 2009[84]; Chousos et al., 2010[16]; Mahdi et al., 2018[49]); seven studies were conducted only in men (Watts et al., 1996[80]; Avogaro et al., 1997[3]; O'Brien et al., 1997[55]; Steinberg et al., 2000[71]; Van de Ree et al., 2001[75]; Kingwell et al., 2003[44]; Shemyakin et al., 2012[67]); one study was conducted only in women (Sivitz et al., 2007[68]), and one study did not specify sex (Steinberg et al., 1996[70]). The mean duration of T2D was 6.6±2.5 years with a range of 3.6 (Watts et al., 1996[80]) to 11 (Mahdi et al., 2018[49]) years. The SNP were administered in brachial artery (Ting et al., 1996[74]; Watts et al., 1996[80]; Williams et al., 1996[81]; Avogaro et al., 1997[3]; O'Brien et al., 1997[55]; Hogikyan et al., 1998[36]; Gazis et al., 1999[26]; Vavuranakis et al., 1999[77]; Butler et al., 2000[13]; Preik et al., 2000[62]; Van de Ree et al., 2001[75]; Van Etten et al., 2002[76]; Vehkavaara and Yki-Järvinen, 2004[78]; Natali et al., 2006[54]; Woodman et al., 2006[83]; Schmiedel et al., 2007[66]; Sivitz et al., 2007[68]; Yeh et al., 2009[84]; Shemyakin et al., 2012[67]; Mahdi et al., 2018[49]), femoral artery (Steinberg et al., 2000[71]), femoral vein (Kingwell et al., 2003[44]), and FMS (Steinberg et al., 1996[70]; Caballero et al., 1999[14]; Sokolnicki et al., 2007[69]; Beer et al., 2008[7]; Brooks et al., 2008[10]; Chousos et al., 2010[16]). The SNP dose range was 0.01 (Caballero et al., 1999[14]) and 10 (Avogaro et al., 1997[3]) µg/min. Characteristics of studies with more details are provided in Table 3(Tab. 3) (References in Table 3: Avogaro et al., 1997[3]; Beer et al., 2008[7]; Brooks et al., 2008[10]; Butler et al., 2000[13]; Caballero et al., 1999[14]; Chousos et al., 2010[16]; Enderle et al., 1998[22]; Furuta et al., 2013[23]; Gazis et al., 1999[26]; Goodfellow et al., 1996[29]; Henry et al., 2004[34]; Hogikyan et al., 1998[36]; Huvers et al., 1997[38]; Kimura et al., 2001[43]; Kingwell et al., 2003[44]; Liu et al., 2018[47]; Mahdi et al., 2018[49]; McVeigh et al., 1992[51]; Natali et al., 2006[54]; O'Brien et al., 1997[55]; Preik et al., 2000[62]; Schmiedel et al., 2007[66]; Shemyakin et al., 2012[67]; Sivitz et al., 2007[68]; Sokolnicki et al., 2007[69]; Steinberg et al., 1996[70], 2000[71]; Tan et al., 2004[73]; Ting et al., 1996[74]; Van de Ree et al., 2001[75]; Van Etten et al., 2002[76]; Vavuranakis et al., 1999[77]; Vehkavaara and Yki-Järvinen, 2004[78]; Watts et al., 1996[80]; Williams et al., 1996[81]; Woodman et al., 2006[83]; Yeh et al., 2009[84]; Yugar-Toledo, 2004[85]). 

The effect of GTN on VSM relaxation has been reported in thirteen studies (McVeigh et al., 1992[51]; Goodfellow et al., 1996[29]; Huvers et al., 1997[38]; Enderle et al., 1998[22]; Vavuranakis et al., 1999[77]; Kimura et al., 2001[43]; Henry et al., 2004[34]; Tan et al., 2004[73]; Woodman et al., 2006[83]; Yeh et al., 2009[84]; Furuta et al., 2013[23]; Yugar-Toledo, 2004[85]; Liu et al., 2018[47]). These studies involved 859 patients with T2D and 583 control subjects. The mean age of T2D patients in these studies was 56.4±7.4 years with a range of 42.7 (Liu et al., 2018[47]) to 70 (Kimura et al., 2001[43]) years, and the mean age of control subjects was 52.8±8.6 years with a range of 42 (Furuta et al., 2013[23]) to 68 (Henry et al., 2004[34]) years. Eleven studies included both sexes (McVeigh et al., 1992[51]; Goodfellow et al., 1996[29]; Huvers et al., 1997[38]; Enderle et al., 1998[22]; Vavuranakis et al., 1999[77]; Kimura et al., 2001[43]; Henry et al., 2004[34]; Tan et al., 2004[73]; Woodman et al., 2006[83]; Yeh et al., 2009[84]; Furuta et al., 2013[23]); one study was conducted only in women (Liu et al., 2018[47]), and one study did not specify sex ( Yugar-Toledo, 2004[85]). The mean duration of T2D was 7.7±5.2 years with a range of 3.8 (Goodfellow et al., 1996[29]) to 19.7 (Furuta et al., 2013[23]) years. The GTN was administered in the brachial artery (McVeigh et al., 1992[51]; Goodfellow et al., 1996[29]; Huvers et al., 1997[38]; Enderle et al., 1998[22]; Kimura et al., 2001[43]; Henry et al., 2004[34]; Tan et al., 2004[73]; Woodman et al., 2006[83]; Yeh et al., 2009[84]; Furuta et al., 2013[23]; Yugar-Toledo, 2004[85]; Liu et al., 2018[47]) and coronary artery (Vavuranakis et al., 1999[77]). The range of doses of GTN used were 68 (McVeigh et al., 1992[51]) and 400 (Goodfellow et al., 1996[29]) µg/min. Characteristics of studies with more details are provided in Table 3(Tab. 3).

### Meta-analyses: ET-independent VSM relaxation 

**Figure 5:**
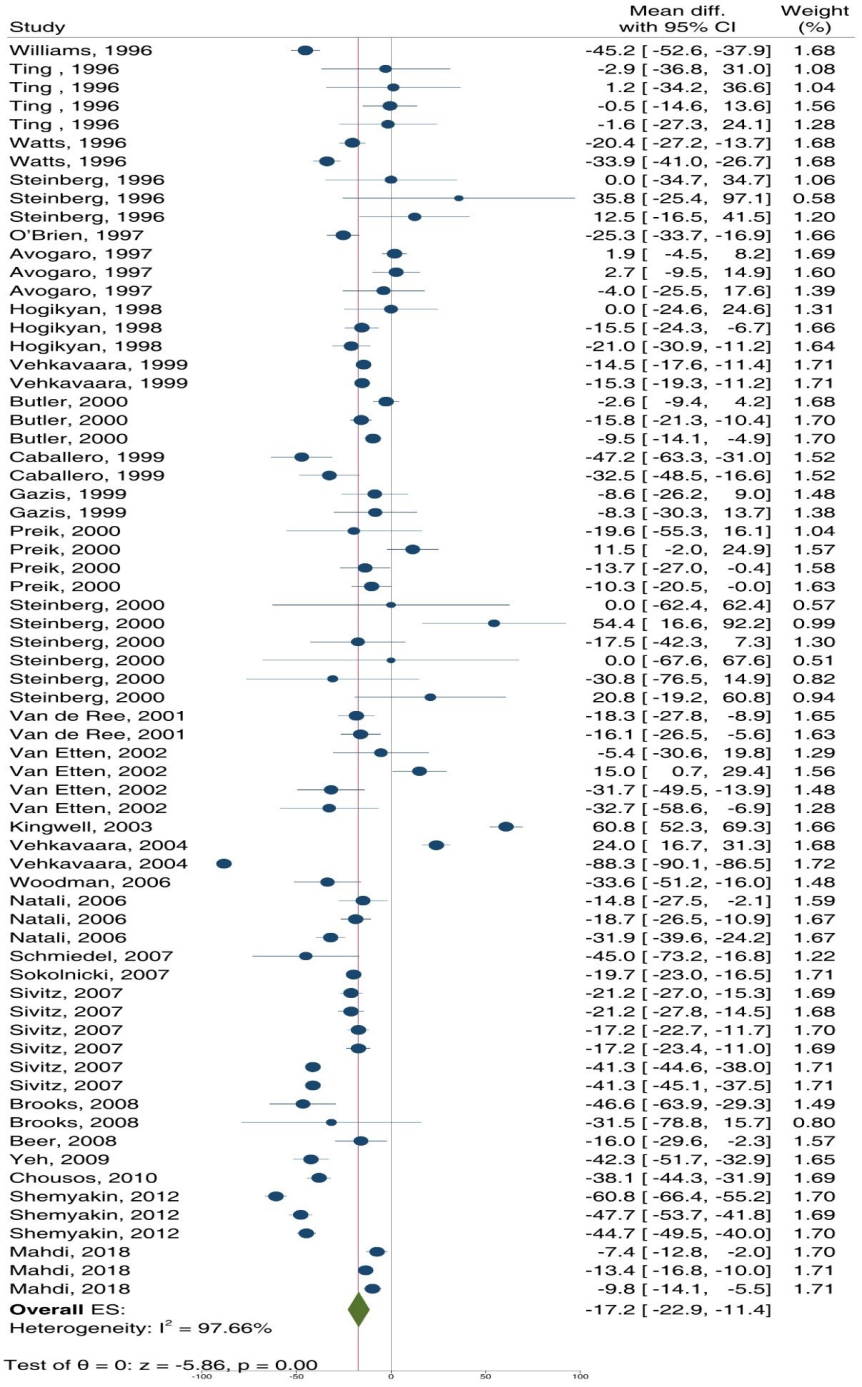
Overall effect size (ES) of endothelium (ET)-independent vascular relaxation in response to sodium nitroprusside (SNP) in type 2 diabetic patients compared to controls. CI, confidence interval.

**Figure 6:**
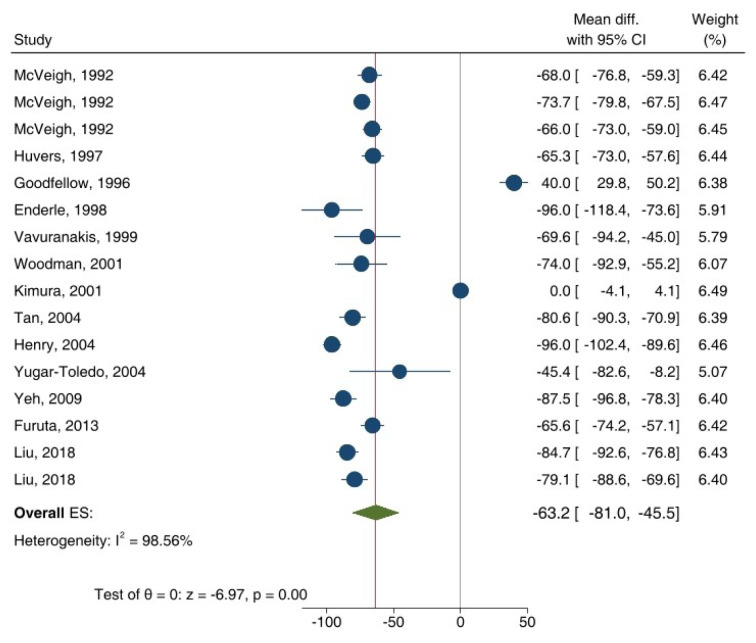
Overall effect size (ES) of endothelium (ET)-independent vascular relaxation in response to glyceryl trinitrate (GTN) in type 2 diabetic patients compared to controls. CI, confidence interval

ET-independent VSM relaxation in response to SNP (overall ES = -17.2 %, 95 % CI: -35.2, -22.7; p<0.001) (Figure 5(Fig. 5); References in Figure 5: Avogaro et al., 1997[3]; Beer et al., 2008[7]; Brooks et al., 2008[10]; Butler et al., 2000[13]; Caballero et al., 1999[14]; Chousos et al., 2010[16]; Gazis et al., 1999[26]; Hogikyan et al., 1998[36]; Kingwell et al., 2003[44]; Mahdi et al., 2018[49]; Natali et al., 2006[54]; O'Brien et al., 1997[55]; Preik et al., 2000[62]; Schmiedel et al., 2007[66]; Shemyakin et al., 2012[67]; Sivitz et al., 2007[68]; Sokolnicki et al., 2007[69]; Steinberg et al., 1996[70], 2000[71]; Ting et al., 1996[74]; Van de Ree et al., 2001[75]; Van Etten et al., 2002[76]; Vavuranakis et al., 1999[77]; Vehkavaara and Yki-Järvinen, 2004[78]; Watts et al., 1996[80]; Williams et al., 1996[81]; Woodman et al., 2006[83]; Yeh et al., 2009[84]) and GTN (overall ES = -63.2 %, 95 % CI: -81.0, -45.5; p<0.001) (Figure 6(Fig. 6); References in Figure 6: Enderle et al., 1998[22]; Furuta et al., 2013[23]; Goodfellow et al., 1996[29]; Henry et al., 2004[34]; Huvers et al., 1997[38]; Kimura et al., 2001[43]; Liu et al., 2018[47]; McVeigh et al., 1992[51]; Tan et al., 2004[73]; Vavuranakis et al., 1999[77]; Woodman et al., 2006[83]; Yeh et al., 2009[84]; Yugar-Toledo, 2004[85]) decreased in T2D patients compared to controls. In addition, high heterogeneity was observed in these analyses, indicated by I² values of 97.7 % (p<0.001) and 98.6 % (p<0.001) for SNP and GTN, respectively. Overall, ET-independent VSM relaxation in response to SNP/GTN in T2D patients decreased by 25.8 % (95 % CI: -32.7, -18.8; p<0.001).

### Meta-regressions, subgroup analysis, and publication bias: ET-independent VSMrelaxation 

ET-independent VSM relaxation in response to SNP was affected by the doses of SNP (p= 0.084, B=-1.36; Supplementary Figure 1E) and the duration of T2D (p= 0.047, B=-3.36; Supplementary Figure 1F); higher doses of SNP and longer duration of T2D were associated with a higher WMD (higher impairment in response to SNP) of VSM relaxation between T2D patients and controls. ET-independent VSM relaxation in response to GTN has not been affected by GTN doses (p=0.942, B = -0.006; Supplementary Figure 1G) but affected by the duration of T2D (p=0.071, B = -0.2631.96; Supplementary Figure 1H); longer durations of T2D were associated with a higher WMD (higher impairment in response to GTN) of VSM relaxation between T2D patients and controls. 

The test of group differences was statistically significant between the subgroups for the type of vessels in studies that used SNP (p=0.045) but not for GTN (p=0.809) (Table 4(Tab. 4)).

The funnel plot for the mean difference in the VSM function of studies included in the meta-analysis was asymmetrical (Supplementary Figure 2C for SNP and 2D for GTN). However, Egger's test detected that the observed asymmetry was not due to publication bias (p=0.315, p=0.879 for SNP and GTN, respectively).

## Discussion

In this study, following our previous narrative review study (Bahadoran et al., 2023[5]), we systematically reviewed and meta-analyzed data from 44 non-randomized clinical trials (with 68, 16, 30, and 50 study arms for ACh, MTH, SNP, and GTN, respectively). These trials compared ET-dependent and -independent VSM relaxation in T2D patients and controls. Our analysis revealed a decrease in ET-dependent VSM relaxation in T2D patients by ~40 %, with a greater decrease for MTH (~55 %) compared to ACh (~30 %). Similarly, ET-independent VSM relaxation in T2D patients decreased by ~25 %. We observed a greater decrease for GTN (~63 %) compared to SNP (~17). Therefore, the current, up-to-date meta-analysis confirmed the results of Montero et al. (2013[52]) and, for the first time, showed that impairment of ET-dependent and -independent VSM relaxation in T2D depends on the type and dose of vasodilators used. In addition, ET-dependent (MTH) and ET-independent (SNP/GTN) VSM relaxation in T2D patients are affected by the duration of T2D and the type of vessels studied. 

Our results showed that ET-dependent (ACh/MTH) VSM relaxation in T2D patients decreased by ~40 %, and the decrease was higher in response to MTH (~55 %) compared to ACh (~30 %), a difference of about two-fold. Although both ACh and MTH are non-selective cholinergic agonists with similar affinity for muscarinic receptors, ACh exhibits significantly lower potency in inducing vasodilatation (-log EC_50_ = 6.43 vs. 7.24 mol/L) (Bruning et al., 1995[11], 1996[12]). This difference can be attributed to the rapid degradation of ACh by cholinesterases. Consequently, MTH has a longer-lasting effect, leading to a more pronounced (10-fold) vasodilator response (Bruning et al., 1995[11], 1996[12]). 

Our results showed that ET-independent (SNP/GTN) VSM relaxation in T2D patients decreased by ~25 %, and this decrease was higher in response to GTN (~63 %) compared to SNP (~17 %) in T2D patients. It has been reported that GTN can be more potent than SNP under specific conditions, exhibiting ~1,000-fold higher vasodilatory activity in certain conditions in rat chest vessels (Badejo et al., 2010[4]) and in human coronary arteries (He and Yang, 1997[32]). Unlike the consistent findings on impaired ET-dependent VSM relaxation, studies investigating ET-independent VSM relaxation in T2D patients in response to GTN and SNP have yielded conflicting results. Some studies reported a decrease of 6-42 % in SNP (Morris et al., 1995[53]; Watts et al., 1996[80]; Preik et al., 2000[62]) and GTN (McVeigh et al., 1992[51]; Vavuranakis et al., 1999[77]) induced VSM relaxation, while others show preserved VSM relaxation (Karasu et al., 1995[39]; Steinberg et al., 1996[71]; Heitzer et al., 2000[33]) in T2D patients. These discrepancies likely arise from several factors, including the presence of diabetes complications (Avogaro et al., 1997[3]), disease severity and duration (Natali et al., 2006[54]), and T2D management (Bock et al., 2021[8]). These factors are estimated to explain 32-37 % of the variation observed in ET-independent VSM relaxation (Caballero et al., 1999[14]; Bahadoran et al., 2023[5]). Interestingly, ET-independent VSM relaxation remains preserved in the brachial artery of T2D patients without complications (Avogaro et al., 1997[3]). Additionally, reduced SNP-induced vasodilation was only observed in neuropathic T2D patients (Pitei et al., 1997[61]). Studies also report a progressive decrease in ET-independent VSM relaxation from 3 % in insulin-sensitive patients to 27 % in insulin-resistant patients (Natali et al., 2006[54]). Furthermore, insulin therapy for 6 months and a 3.5-year follow-up can improve SNP-induced vasodilation in T2D patients (Vehkavaara and Yki-Järvinen, 2004[78]).

Results of meta-regression analysis showed that the duration of T2D affects MTH and SNP/GTN-induced VSM relaxation in T2D patients. For MTH (with a maximum T2D duration of 4 years), longer T2D was associated with a smaller difference in VSM relaxation in T2D patients compared to controls. In contrast, SNP/GTN studies (with a maximum T2D duration of 20 years) showed a greater difference in VSM relaxation in T2D patients than in controls. Importantly, this difference in study duration between MTH and SNP/GTN studies may explain the opposing findings. 

Decreased NO production by the ET and impaired responsiveness of VSMs to NO have been reported in the late stages of T2D (Bahadoran et al., 2023[5]). Early T2D stages, however, may exhibit increased NO production (Adela et al., 2015[1]), potentially as a compensatory mechanism. Over time, this ability diminishes, leading to a state of vascular NO deficiency in established T2D (Pieper, 1998[59]; Salvatore et al., 2022[64]). In support, ACh-induced NO release in the thoracic aorta of rats enhanced at 12 weeks but decreased at 36 weeks (Kobayashi et al., 2004[45]). A progressive decreased endothelial NO synthase sensitivity to ACh was also shown to be worsened with the diabetes duration in rats; EC_50_ for ACh in diabetic arteries was increased from 13.5 nM after 6 weeks to 100 nM after 24 weeks of diabetes (Diederich et al., 1994[19]). 

Our subgroup analysis showed that ET-dependent (ACh-induced) VSM relaxation in T2D patients was significantly lower (higher impairment) than controls when using brachial and femoral arteries (macrocirculation) compared to FMS. Conversely, ET-independent (SNP-induced) VSM relaxation was significantly lower in FMS compared to macrocirculation. This finding aligns with a meta-analysis reporting higher impairment of ET-independent VSM relaxation in microcirculation versus macrocirculation in T2D patients compared to controls (Montero et al., 2013[52]). Evidence suggests that small coronary arteries might be more susceptible to T2D-induced vascular NO resistance compared to large arteries (Oltman et al., 2006[56]). This could be due to variations in NO production capacity and responsiveness to NO's vasodilatory effects between different blood vessel types (Bahadoran et al., 2023[5]). These observations may imply that different vessels probably exhibit diverse phenotypes of NO resistance in T2D. 

**Figure 7:**
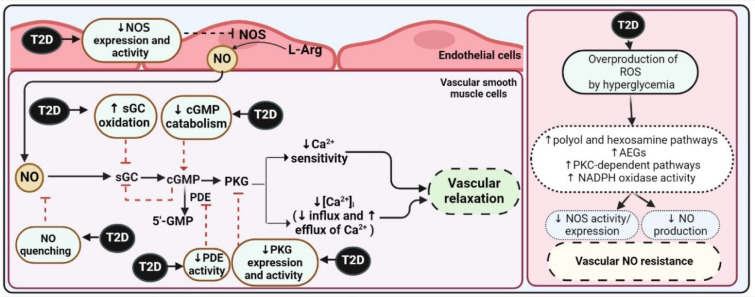
Type 2 diabetes (T2D) impairs both ET-dependent and -independent vascular smooth muscle (VSM) relaxation by affecting nitric oxide (NO) action on VSM relaxation. The vascular hypo-responsiveness to NO is evident at the receptor and post-receptor level. AEGs, advanced glycation end products; cGMP, cyclic guanosine monophosphate; L-Arg, l-arginine; NADPH, nicotinamide adenine dinucleotide phosphate; NOS, Nitric oxide synthase; PDE, phosphodiesterase; PKG, protein kinase G; PKC, protein kinase C; ROS, reactive oxygen species; sGC, soluble guanylate cyclase

In summary, both ET-dependent and ET-independent VSM relaxation are impaired in T2D. Various studies, including a recent review by our group, have investigated the mechanisms underlying this impairment (Bahadoran et al., 2023[5]). Although, the exact mechanisms remain unclear; hyperglycemia is thought to play a significant role. Hyperglycemia increases the production of reactive oxygen species (ROS) in the vessel wall, triggering a series of events: activation of the polyol and hexosamine pathways, formation of advanced glycation end products (AGEs), activation of protein kinase C (PKC) pathways, and increased nicotinamide adenine dinucleotide phosphate (NADPH) oxidase activity. These events collectively impair NO-induced VSM relaxation, resulting in vascular hypo-responsiveness to NO (Figure 7(Fig. 7)). In ET cells, T2D primary blocks nitric oxide synthase (NOS) activity and expression, leading to lower NO bioavailability in VSM cells. In VSM cells, one key mechanism is the impairment of the NO-cyclic guanosine monophosphate (cGMP)-protein kinase G (PKG) pathway at both the receptor and post-receptor levels. This may occur through quenching of NO and desensitization of soluble guanylate cyclase (sGC) at the receptor level (Witte et al., 2002[82]; Goulopoulou et al., 2015[30]), and through decreased cGMP degradation and altered activity and expression of PKG at the post-receptor level (Chirkov and Horowitz, 2007[15]; Russo et al., 2008[63]). More details on the mechanisms of vascular relaxation impairment in T2D can be found elsewhere (Bahadoran et al., 2023[5]).

Our study has some limitations; first, we identified significant heterogeneity, meaning that the results of the included studies varied considerably. Egger's test detected that the observed heterogeneity was not due to publication bias. While subgroup analyses suggested vessel type as the potential source of heterogeneity, the uneven distribution of studies across subgroups limits definitive conclusions. Additionally, the studies themselves varied in design, patient numbers, doses of vasodilators, and T2D duration. These design variations might contribute to the observed heterogeneity. Second, another limitation is that our study did not account for the potential confounding effects of diabetic complications or medications taken by diabetic patients. One strength of our study is that we considered baseline values of VSM in brachial and femoral arteries, as well as FMS of diabetic patients. This analysis revealed a 13 % lower baseline VSM (indicating lower baseline blood flow) in T2D patients compared to controls within the studies included in our meta-analysis. This finding highlights a potentially important factor that was not considered by Montero et al. (2013[52]). This allows for a clearer interpretation of results as we assess the change in relaxation due to T2D rather than simply comparing the values of diabetic and control groups. In addition, the weight of the included studies was balanced across different analyses. This suggests the overall ES is not driven by specific studies and is unlikely to be a false-positive result.

## Conclusion

Our meta-analysis of clinical trials investigated the effects of T2D on VSM relaxation. The results showed significant reductions in both ET-dependent (~40 %) and ET-independent (~25 %) VSM relaxation. The decrease was more pronounced for MTH (~55 %) compared to ACh (~30 %) and for GTN (~63 %) compared to SNP (~17 %). These findings suggest that dysfunction of both ET and VSM contributes to impaired VSM relaxation in T2D patients. 

Results obtained from ET-dependent (MTH) and ET-independent (SNP/GTN) VSM relaxation in T2D patients may be affected by both the type of studied vessels and the duration of T2D. This knowledge may be relevant for developing treatments specifically targeting different blood vessels in T2D. Considering the diverse phenotypes of NO resistance in different vessels may help develop specific vessel-targeted drug delivery platforms to overcome vascular NO resistance in T2D. Pharmacological approaches such as angiotensin-converting enzyme inhibitors, the anti-anginal agent perhexiline, insulin, statins, nitrite, and sGC activators have been proposed to attenuate vascular NO resistance by decreasing ROS production and increasing eNOS expression and activity (Chirkov and Horowitz, 2007[15]; Velagic et al., 2020[79]). NO-releasing drugs may not affect microcirculation, which has a higher impairment of ET-independent VSM relaxation and is relatively nonresponsive to NO compared to macrocirculation. In these cases, the use of nitroxyl donors, such as Angeli's salt, has been suggested as an effective treatment for overcoming NO resistance in T2D, where NO-releasing drugs do not act effectively (Velagic et al., 2020[79]). Angeli's salt can effectively bypass NO resistance in vessels that become relatively nonresponsive to NO in T2D by affecting on distinct molecules (e.g., thiol residues) and pathways [cyclic AMP (cAMP)-protein kinase A (PKA)]. Moreover, the duration of T2D should be considered when designing treatment strategies for vascular complications. Because various vessels have different capacities of NO production (Bohlen, 2015[9]) and respond heterogeneously to the vasodilatory action of NO, likely due to their different morphologies, functions, and diverse receptor and ion channel populations (Clark and Fuchs, 1997[17]; Gao et al., 1998[25]; Houghton et al., 1998[37]). Vascular NO resistance seems to progress over time, with NO production increasing in the initial stages of T2D (within 5 years of onset) and decreasing in established T2D (Pieper, 1998[59]; Salvatore et al., 2022[64]). Upregulated NOS expression/activity may explain the elevated NO production in the initial stages of T2D (Kazuyama et al., 2009[41]). Although it needs to be proved, small coronary arteries appear to be affected by T2D-induced vascular NO resistance earlier and to a greater extent compared to mesenteric resistance arteries and large elastic vessels like the aorta (Oltman et al., 2006[56]). These observations suggest that some treatments, such as NO-releasing drugs and activators of NOS expression, may not affect impairment in VSM relaxation in the early stage T2D. Management of hyperglycemia, insulin resistance and oxidative stress are essential treatments during this stage (Bahadoran et al., 2023[5]).

## Notes

Khosrow Kashfi and Asghar Ghasemi (Endocrine Physiology Research Center, Research Institute for Endocrine Sciences, Shahid Beheshti University of Medical Sciences, Tehran, Iran; E-mail: Ghasemi@sbmu.ac.ir) contributed equally as corresponding author.

## Declaration

### Acknowledgments and funding information

This study was supported by a grant from Shahid Beheshti University of Medical Sciences.

### Declaration of competing interest 

The authors declare that they have no competing interests.

### Authorships

Sajad Jeddi, Zahra Bahadoran, and Parvin Mirmiran contributed to the screening and quality assessment of studies and data extraction. Sajad Jeddi, Zahra Bahadoran, Parvin Mirmiran, Khosrow Kashfi, and Asghar Ghasemi contributed to the literature review and wrote the article. Sajad Jeddi, Khosrow Kashfi, and Asghar Ghasemi provided critical revision and final approval of the finalized manuscript. All authors have read and approved the final manuscript. 

## Supplementary Material

Supplementary information

## Figures and Tables

**Table 1:**
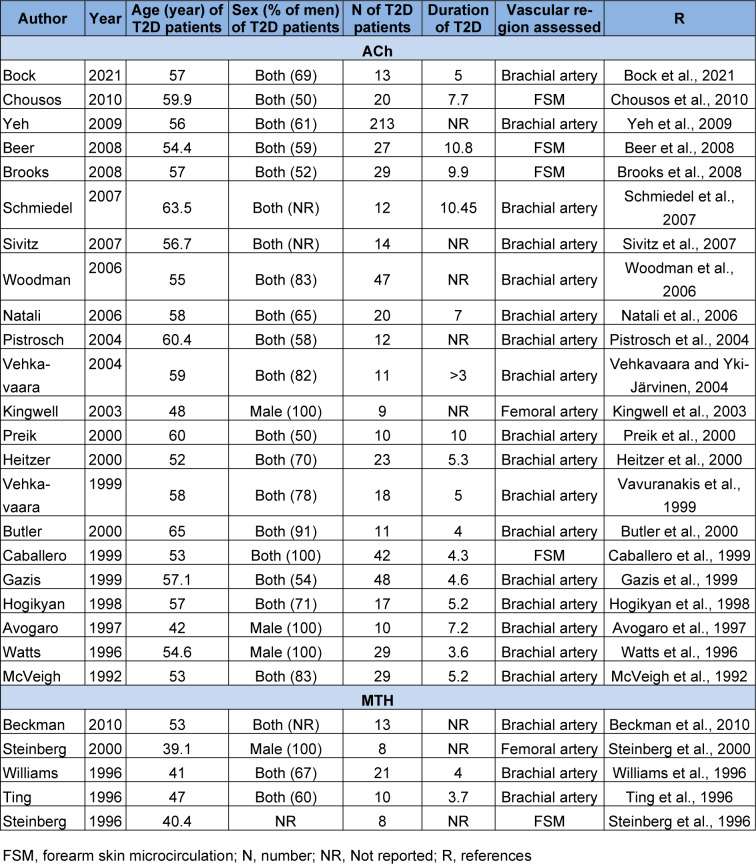
Characteristic of studies that reported endothelium-dependent vascular smooth muscle (VSM) relaxation in patients with type 2 diabetes (T2D) in response to acetylcholine (ACh) and methacholine (MTH)

**Table 2:**
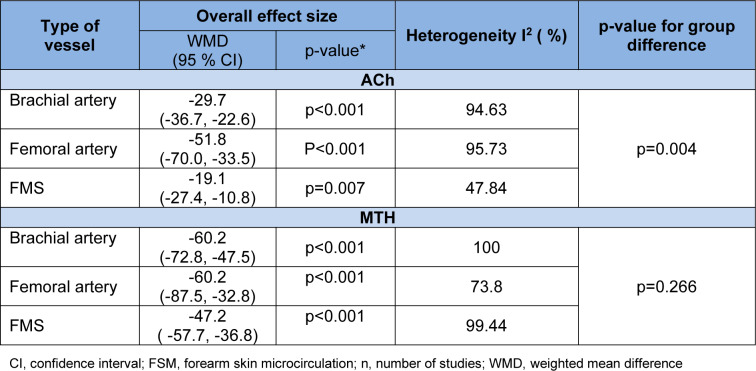
Subgroup analysis of endothelium (ET)-dependent vascular smooth muscle (VSM) relaxation in response to acetylcholine (ACh) and methacholine (MTH) in patients with type 2 diabetes (T2D) compared to controls

**Table 3:**
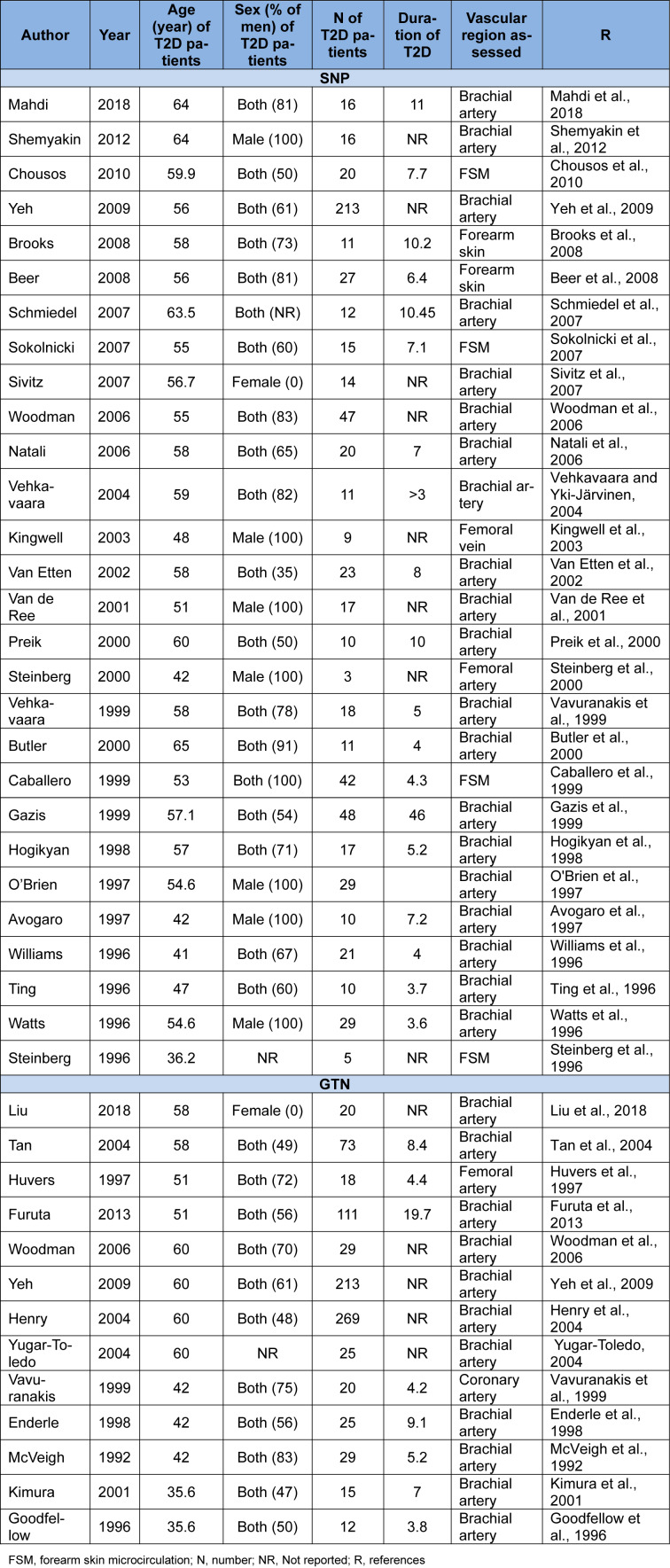
Characteristic of studies that reported endothelium (ET)-independent vascular smooth muscle (VSM) relaxation in patients with type 2 diabetes (T2D) to sodium nitroprusside (SNP) and glyceryl trinitrate (GTN)

**Table 4:**
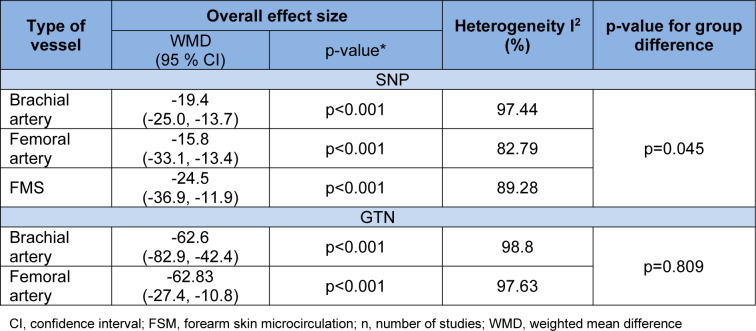
Subgroup analysis of endothelium (ET)-independent vascular smooth muscle (VSM) relaxation in response to sodium nitroprusside (SNP) and glyceryl trinitrate (GTN) in patients with type 2 diabetes (T2D) compared to controls

**Figure 1:**
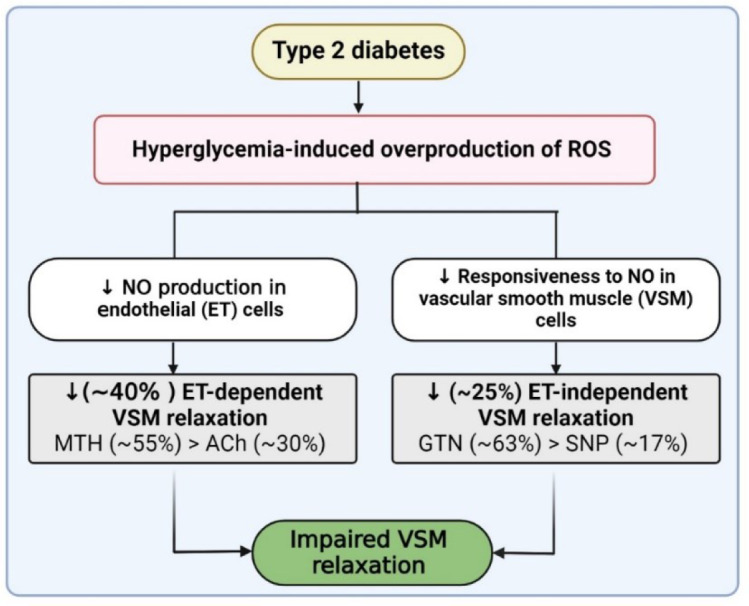
Graphical abstract. Type 2 diabetes decreased both ET-dependent (~40 %) and ET-independent (~25 %) vascular smooth muscle (VSM) relaxation that was more pronounced for MTH (~55 %) compared to ACh (~30 %) and for GTN (~63 %) compared to SNP (~17 %). ACh, acetylcholine; GTN, glyceryl trinitrate; MTH, methacholine; NO, nitric oxide; ROS, reactive oxygen species; SNP, sodium nitroprusside
