# Supplementary material for: Impaired vascular relaxation in type 2 diabetes: A systematic review and meta-analysis
Source: EXCLI J. 2024 Jul 9;23:937–59. doi: 10.17179/excli2024-7330 (PMC11382252; doi:10.17179/excli2024-7330)
Supplement: Supplementary information [file EXCLI-23-937-s-001.pdf]

EXCLI Journal 2024;23:937-959 – ISSN 1611-2156
Received: May 01, 2024, accepted: July 01, 2024, published: July 09, 2024

Supplementary information to:

Review article:

IMPAIRED VASCULAR RELAXATION IN TYPE 2 DIABETES: A SYSTEMATIC REVIEW AND META-ANALYSIS

Sajad Jeddi[1], Zahra Bahadoran[2], Parvin Mirmiran[2], Khosrow Kashfi[3*], Asghar Ghasemi[1*]

[1] Endocrine Physiology Research Center, Research Institute for Endocrine Sciences, Shahid Beheshti University of Medical Sciences, Tehran, Iran
[2] Nutrition and Endocrine Research Center, Research Institute for Endocrine Sciences, Shahid Beheshti University of Medical Sciences, Tehran, Iran
[3] Department of Molecular, Cellular, and Biomedical Sciences, Sophie Davis School of Biomedical Education, City University of New York School of Medicine, NY, USA

* **Corresponding authors:** Khosrow Kashfi, PhD, FRSC, FRSB, Department of Molecular, Cellular, and Biomedical Sciences, Sophie Davis School of Biomedical Education, City University of New York School of Medicine, NY, USA NY, 10031, USA.
E-mail: Kashfi@med.cuny.edu
Asghar Ghasemi, PhD, Endocrine Physiology Research Center, Research Institute for Endocrine Sciences, Shahid Beheshti University of Medical Sciences, Tehran, Iran.
E-mail: Ghasemi@sbmu.ac.ir

https://dx.doi.org/10.17179/excli2024-7330

This is an Open Access article distributed under the terms of the Creative Commons Attribution License (http://creativecommons.org/licenses/by/4.0/).

EXCLI Journal 2024;23:937-959 – ISSN 1611-2156
Received: May 01, 2024, accepted: July 01, 2024, published: July 09, 2024

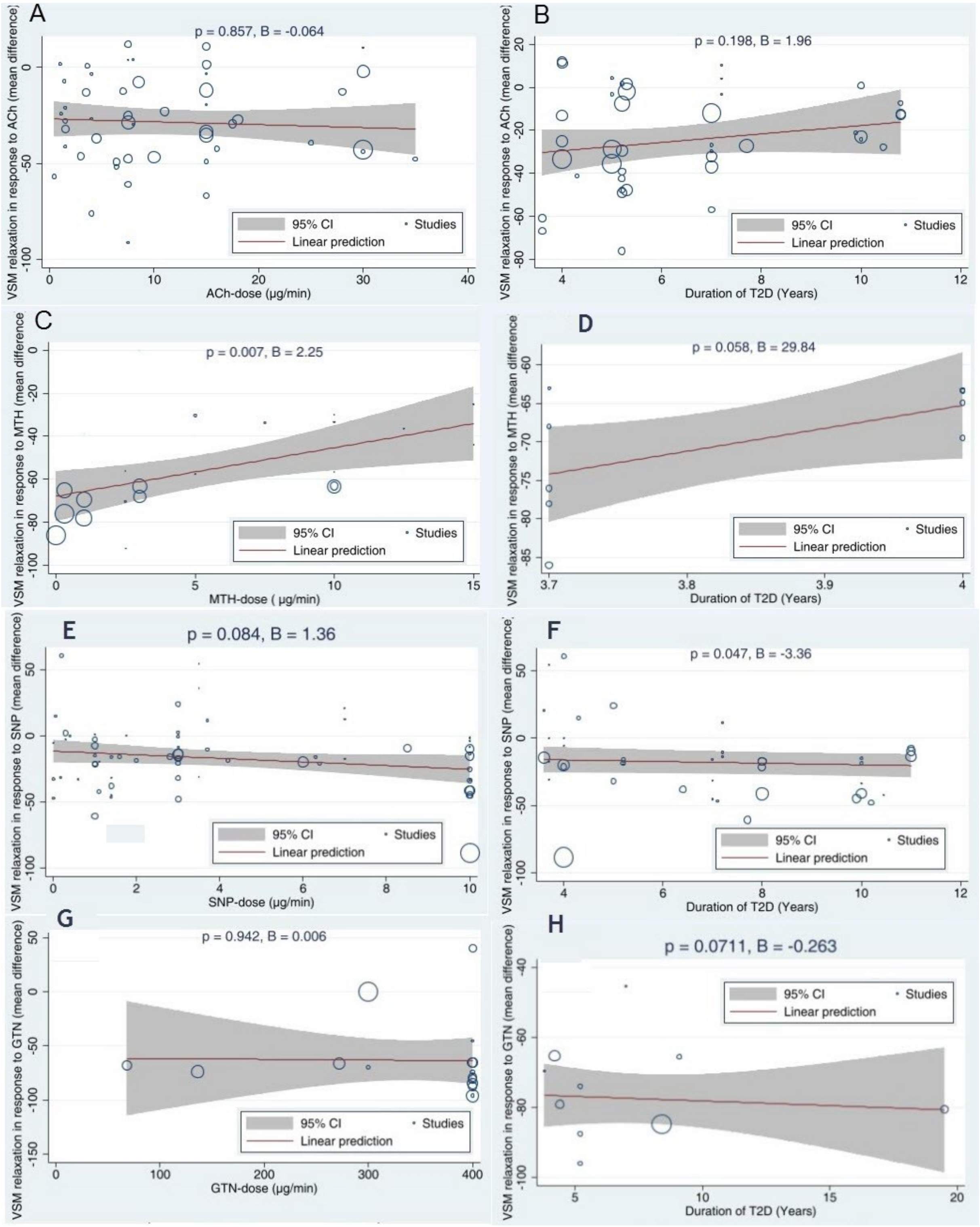

Supplementary Figure 1: Meta-regression analysis explores the association between weighted mean differences in vascular smooth muscle (VSM) relaxation and four factors: acetylcholine (ACh) dose (**A**), duration of T2D in patients receiving ACh (**B**), methacholine (MTH) dose (**C**), and duration of T2D in patients receiving MTH (**D**), sodium nitroprusside (SNP) dose (**E**), duration of type 2 diabetes (T2D) in patients receiving SNP (**F**), glyceryl trinitrate (GTN) dose (**G**), and duration of T2D in patients receiving GTN (**H**). CI, confidence interval

EXCLI Journal 2024;23:937-959 – ISSN 1611-2156
Received: May 01, 2024, accepted: July 01, 2024, published: July 09, 2024

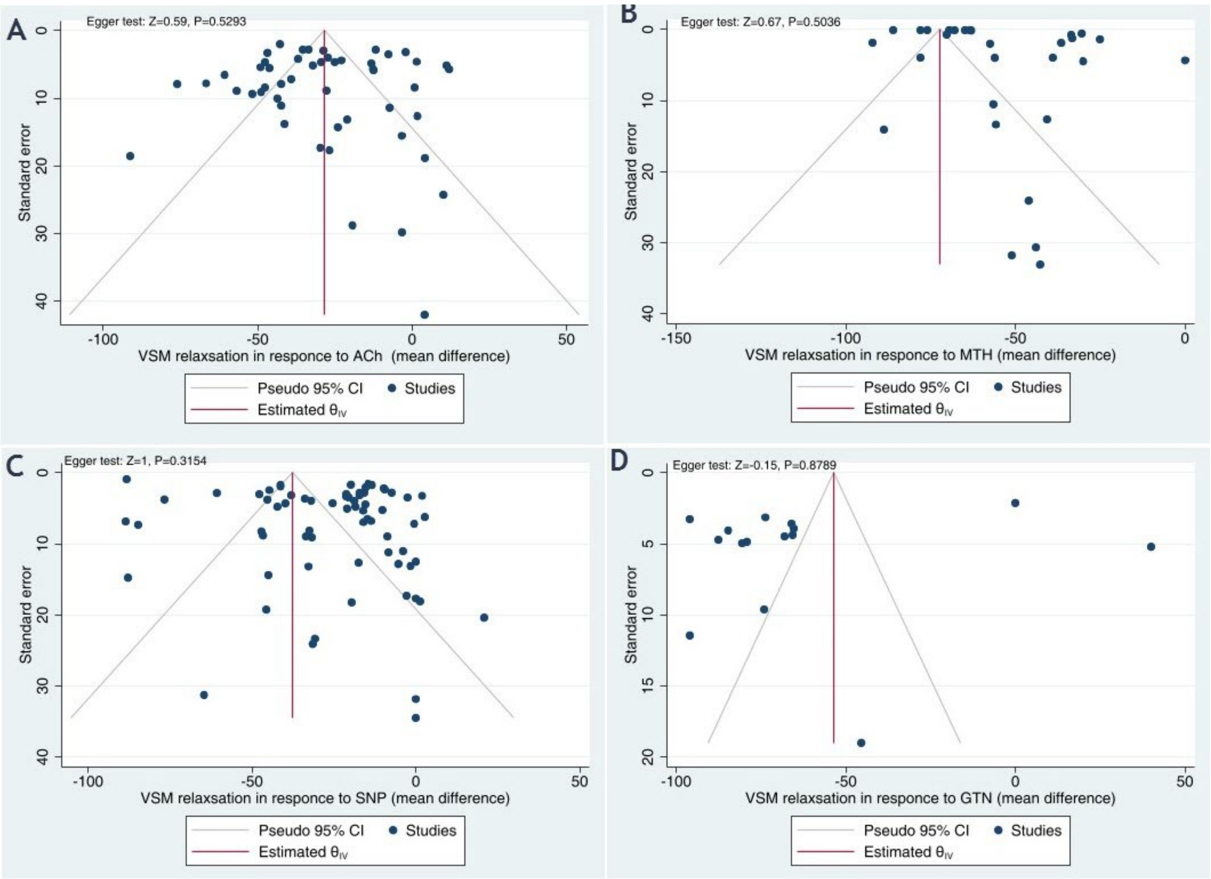

Supplementary Figure 2: Funnel plot of studies included ACh (**A**), MTH (**B**), SNP (**C**), and GTN (**D**) in the meta-analysis. CI, confidence interval
